# Knowledge Gaps in the Fetal to Neonatal Transition of Infants With a Congenital Diaphragmatic Hernia

**DOI:** 10.3389/fped.2021.784810

**Published:** 2021-12-14

**Authors:** Philip L. J. DeKoninck, Emily J. J. Horn-Oudshoorn, Ronny Knol, Kelly J. Crossley, Irwin K. M. Reiss

**Affiliations:** ^1^Department of Obstetrics and Gynecology, Division Fetal Medicine, Erasmus MC University Medical Center—Sophia Children's Hospital, Rotterdam, Netherlands; ^2^The Ritchie Centre, Hudson Institute of Medical Research, Melbourne, VIC, Australia; ^3^Department of Pediatrics, Division of Neonatology, Erasmus MC University Medical Center—Sophia Children's Hospital, Rotterdam, Netherlands; ^4^Department of Obstetrics and Gynaecology, School of Clinical Sciences, Monash University, Melbourne, VIC, Australia

**Keywords:** congenital diaphragmatic hernia, birth, cord clamping, neonatal transition, oxygen, respiratory monitoring

## Abstract

Clinical research for infants born with a congenital diaphragmatic hernia (CDH) has until recently mainly focused on advances in prenatal and postnatal treatment. However, during the early perinatal transition period there are major physiological adaptations. For most infants these changes will happen uneventfully, but for CDH infants this marks the beginning of serious respiratory complications. In recent years, there is emerging evidence that the clinical management during the perinatal stabilization period in the delivery room may influence postnatal outcomes. Herein, we discuss major knowledge gaps and novel concepts that aim to optimize fetal to neonatal transition for infants with CDH. One such novel and interesting approach is performing resuscitation with an intact umbilical cord, the efficacy of this procedure is currently being investigated in several clinical trials. Furthermore, close evaluation of neonatal physiological parameters in the first 24 h of life might provide early clues concerning the severity of lung hypoplasia and the risk of adverse outcomes. We will provide an overview of trending concepts and discuss potential areas for future research.

## Introduction

The management of infants with a congenital diaphragmatic hernia (CDH) is continuously evolving with major improvements in prenatal and postnatal care. Most advances are based on solid scientific evidence using available animal models of CDH prior to translating it into the clinical setting (1). For many of the *in vivo* experiments done in small animals (rabbit, rat and mice models), the endpoint is birth given the lethality of the condition without intensive care. To investigate novel concepts in early postnatal care large animal models (such as the ovine model) are often necessary, yet these experiments are costly and require a dedicated research facility.

Until recently, the transition period defined as the time immediately after birth, has been relatively overlooked. In fact, for a long time the main intervention was to clamp the cord and transfer the infant to the resuscitation table for further stabilization as soon as possible (2–4). On the other hand, major physiological adaptations occur during this immediate postnatal period and a complicated course may effect long term outcomes (5).

In the past decades there has been tremendous effort invested in optimizing the perinatal stabilization period for infants born preterm with immature lungs or those that may undergo problematic fetal to neonatal transition; such as due to birth asphyxia or in case of an elective cesarean section (6, 7). Our knowledge of the physiology underpinning the changes at birth has dramatically improved and novel concepts concerning the timing of cord clamping, oxygen management and the type and level of respiratory support required were introduced to clinical practice (7, 8).

**Figure 1:**
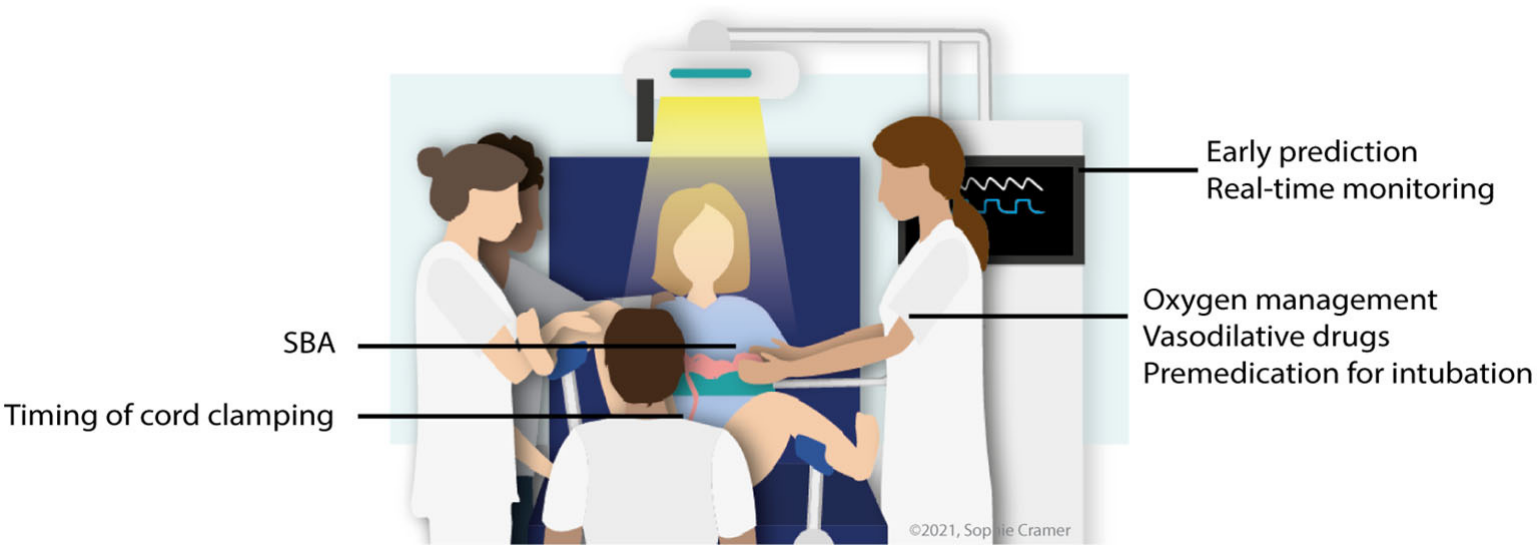
Overview of potential research areas concerning perinatal stabilization of CDH infants. SBA, spontaneous breathing approach. Adapted from Knol et al., (15) with permission of the illustrator.

Some of these approaches are now being evaluated in large clinical trials, but the promising preliminary results have also inspired researchers to investigate their effectiveness for conditions that affect *in-utero* lung development, such as CDH (9–14). Research about neonatal transition for infants with a CDH is rapidly developing, in this literature review we describe new insights and we discuss knowledge gaps for future research (Figure 1).

## Intact Cord Resuscitation

For most infants, adequate gas-exchange is promptly established after birth, i.e., within the first breaths, by rapid clearance of lung liquid resulting in aeration of the lungs. However, infants born with lung hypoplasia have a reduced liquid clearance rate, which is proportional to the lung size and thus reduces the infant's ability to aerate its lungs (16). This problem is likely a reflection of a simplified distal airway architecture and as such a reduced cross sectional area for moving lung liquid into the interstitial tissues. Furthermore, hypoplastic lungs generally have a higher elastic recoil (stiffer) demonstrated by a lower dynamic lung compliance (10, 16, 17).

Lung aeration is considered a key factor in driving vasodilation of the pulmonary vasculature and thereby increasing pulmonary blood flow (18). Apart from establishing adequate gas-exchange, lung aeration is essential for a smooth cardiovascular transition from a fetus to a newborn. Immediately after umbilical cord clamping there is a sudden increase in peripheral vascular resistance and at the same instant venous return to the left atrium via the ductus venosus and foramen ovale stops. In the hypoplastic lung, pulmonary vascular resistance remains high and therefore adequate left venous return is not rapidly restored, whereas in normally developed lungs venous return is established within the first breaths (10, 18). A delayed restoration of venous return translates in a sudden decrease in cardiac output (30–50% reduction) and neonatal hypoxemia, which is considered a risk factor for developing persistent pulmonary hypertension (9). Furthermore, the impaired vascular relaxation forces higher pulmonary perfusion pressures to maintain adequate pulmonary blood flow. We have recently shown that in a lamb CDH model, after an initial improvement in pulmonary vascular resistance, this short period of exposure to higher driving pressures may be a trigger for developing pulmonary hypertension at a later stage (9). This observation could be the physiological explanation of the so-called ‘honeymoon’ period, a transient time of clinical stability, that is observed in some infants with CDH (19).

In recent years, the importance of delaying cord clamping until after lung aeration (and adequate left venous return) has gained momentum, specifically in preterm infants born with immature lungs. Likewise, there have recently been two feasibility studies evaluating this approach for infants born with CDH (11, 12). An important consideration is the need to provide mechanical ventilation to the neonate in close proximity to the mother whilst the integrity of the umbilical cord remains intact. A mobile resuscitation trolley is required for this approach to be successful and several alternatives are currently commercially available (20). These trolleys have inherent limitations and advantages, which are important to consider when implementing intact cord resuscitation, as well as the financial aspect given considerable differences in acquisition costs.

Both studies, although small sample sizes (*n* = 20), reported good feasibility of 85% and 100%, respectively (11, 12). It is obviously not possible to draw firm conclusions, however both found improved cardiovascular adaptation, resulting in higher blood pressures, less need for cardiac resuscitation and higher Apgar scores (11, 12). These promising findings led to the initiation of two large randomized trials: Congenital Hernia Intact Cord (CHIC, *NCT04429750*) and Physiological-based cord clamping for infants with a Congenital Diaphragmatic Hernia (PinC, *NCT04373902*) (21).

These two trials aim to defer cord clamping until after the infant's lungs are aerated, which is challenging to determine. CO_2_ detectors, respiratory monitors or bedside echocardiography (ductus arteriosus evaluation) could be used for this purpose, but they have inherent technical limitations or are logistically not always feasible in the immediate postnatal setting. Hence, physiological parameters such as heart rate, oxygen saturation and the level of oxygen supplementation are considered as a good alternate proxies for determining lung aeration and the state of the infant's cardiovascular adaptation (9, 21). In the future, with the rapid improvement of bedside respiratory monitors, real-time evaluation of tidal volumes or other lung mechanics might be another way to ascertain adequate lung aeration.

The other challenge is to define a clinically relevant primary outcome. The ultimate endpoint is survival to discharge, however despite efforts to standardize postnatal management, considerable bias due to variations in local management make it difficult to determine the actual benefit of performing intact cord resuscitation. The concern of bias is even more pronounced in multicenter trials, however given the incidence of CDH and the required sample sizes it is almost impossible to investigate this in a single center setting. Consequently, short term outcomes such as Apgar scores (CHIC) and pulmonary hypertension (PinC) were chosen as alternative primary outcomes.

The results of these clinical trials are expected in the next two to three years. Despite differences in methodology, a subsequent meta-analysis using individual participant data might strengthen the scientific evidence physiologically based cord clamping even further.

## Spontaneous Breathing Approach

For most infants with CDH, mechanical ventilation is a double-edged sword: it is essential for survival, but prolonged respiratory support also poses a risk of iatrogenic complications such as ventilator-induced lung injury. On the other hand, a small subset of infants born with a very small diaphragmatic defect, hence mild lung hypoplasia, may not develop severe respiratory insufficiency immediately after birth and thus mechanical ventilation may not be necessary. In fact, prior to the routine use of ultrasound this was probably the group that survived the neonatal period with minimal care and that was only diagnosed at childhood age. Moreover, prompt intubation after birth potentially causes stress and pain for the infant, thereby triggering the development of pulmonary hypertension and impacting neonatal transition. Regardless, the main purpose of routine intubation at birth is to avoid transient hypoxia, which is considered an even more important trigger for pulmonary hypertension (22).

We have recently published our experience of adopting a gentler approach during the initial perinatal stabilization phase by allowing spontaneous breathing in a select subset of patients (23). This approach was only offered for infants with an isolated left-sided CDH, born >35 weeks' gestation and predicted to have a very mild degree of lung hypoplasia. The latter was determined by an observed/expected lung-to-head ratio above 50% and an intra-abdominal position of the liver. In this small study (*n* = 15%), 40% of cases were only intubated at the time of postnatal correction of the diaphragmatic defect, thus required limited respiratory support and were discharged earlier from the intensive care unit. But more importantly, a trial of spontaneous breathing, even unsuccessful, did not appear to impact survival or short-term morbidity (23).

In attempt to improve the success rate of this SBA we have drafted a consensus protocol after several meetings with international experts on CDH management, neonatal resuscitation and fetal/neonatal physiology. One of the recommendations is to start non-invasive respiratory support in these infants, as many of the infants in the above-mentioned series required low flow oxygen supplementation or continuous positive airway pressure (CPAP). However, it is not clear whether the key component to facilitate neonatal transition is either oxygen supplementation, distending airway pressure or a combination of both. The use of positive pressure ventilation is certainly controversial as the main concern is insufflation of the digestive tract, which may impair and limit lung expansion. Therefore, nasal high flow therapy could be an interesting option, because it is relatively easy to position the device, well-tolerated and provides a vehicle for oxygen delivery but also generates distending airway pressure. The level of respiratory support can be adjusted based on the individual needs by changing the flow rate and/or concentration of oxygen delivered. An alternative could be the use of a CPAP mask rather than nasal prongs because it is easier to position. Regardless of the treatment modality used, early insertion of a naso- or orogastric tube with continuous section is advised to avoid stomach distention. The result of these consensus meetings is a proposed algorithm for SBA comparable to what is used in the current neonatal resuscitation guidelines, and this protocol will be published soon.

There is an increasing number of centers that are considering or already have started attempting a trial of spontaneous breathing for infants with mild lung hypoplasia. In the absence of a randomized trial, it is essential to collect the outcome data and given the rareness of the abnormality, a multicenter and international collaboration is the most logical step. To accommodate this, a research consortium was founded consisting of partners all over Europe and Australia: Very mild CDH–Spontaneous Breathing Approach; VeSBA. Outcome data will be collected prospectively in a web-based registry.

## Sedate or Not Sedate?

The majority of infants with a CDH will be intubated immediately after birth. Given the urgency to commence respiratory support in these infants and usually the lack of intravenous access intubation is often done without administering any sedation. The physiological responses to awake intubation of neonates are well-described (24, 25). It can be painful for the infant, translating into markers of acute stress such as increased intracranial and systemic blood pressure, bradycardia and reduced transcutaneous oxygen saturation (24, 25). Furthermore, mediastinal shift and neonatal movements can complicate intubation resulting in a higher stress level for the infant. Therefore, in (semi) elective intubation premedication is considered good standard of care. For CDH infants, the priority is on establishing a secure airway for mechanical ventilation and thus vascular access is often obtained later in the stabilization phase. Alternative options to administer drugs are via the umbilical vein (direct puncture, not via a catheter), buccal or intranasal, however the interval to the onset of effect is potentially longer with the latter two (26, 27). In addition, there is an important knowledge gap when it comes to the optimal treatment regimen (type, dosage) of the premedication (28).

## Oxygen Management

Oxygen supplementation is an essential part of perinatal stabilization of an infant with CDH. The aim is to avoid arterial hypoxemia as it may trigger a vasoactive response and many clinicians will initiate oxygen supplementation with 100% oxygen. After the initial stabilization in the delivery room, oxygen administration is titrated based on the infant's needs targeting a pre-ductal saturation of between 80 and 95% (2). In any case, hyperoxia should be avoided because it also has adverse effects by producing oxygen free radicals. This consideration is certainly important for infants with a relative mild degree of lung hypoplasia, as using 100% oxygen supplementation might be counter effective. Oxidative stress and oxygen free radicals are not only associated with short-term neonatal morbidity but may have long lasting influence on development (29, 30). It has been recently demonstrated that even a brief period of high oxygen exposure may attenuate vasoactive response of the pulmonary vessels to treatments such as inhaled nitric oxide (31).

An alternative approach would be to start stabilization with a reduced oxygen concentration and a stepwise increase or decrease guided by the infant's saturation values (13). This approach is comparable to the resuscitation guidelines for preterm infants (8). In a recent series, the safety of such an alternative approach was evaluated, observing comparable rates of perinatal survival, ECMO use and duration of mechanical ventilation compared to historical CDH controls (13). Moreover, the need of 100% oxygen during the perinatal stabilization period provided an early indication of disease severity and subsequent adverse outcomes (13).

Another important knowledge gap is the use of supplemental oxygen during resuscitation of CDH infants whilst the umbilical cord is still intact. A recent study showed that in preterm lambs, pulmonary blood flow was considerably higher to controls when using 100% oxygen during delayed cord clamping (32). Interestingly, this was not causing systemic hyperoxygenation and hypothetically, the placenta may act as a buffer to reduce the arterial oxygen saturation (32).

The degree of supplemental oxygen exposure at birth may also be diminished by initiating vasodilative treatments already during neonatal resuscitation, such as inhaled nitric oxide, as was recently observed in a small series of preterm infants (33). We speculate that using such an approach for CDH infants may facilitate decreasing pulmonary vascular resistance after birth, thereby preventing high perfusion pressures and potentially avoiding a dysregulated vascular tone of the lung vessels (34). Hypothetically, combining this approach with deferring cord clamping until the lungs are aerated, both appear to have a protective effect on the lung vessels, may have a synergistic effect (9).

## Early Predictors of Adverse Outcome

Infants born with CDH will only face respiratory challenges after birth and consequently it is only at that moment that clinicians can determine the true impact on lung development. Prenatal ultrasound and fetal MRI have proven to be very useful in the individual prediction of prognosis, yet it remains challenging to perform a functional assessment of the lungs (35). More specifically, the occurrence and severity of pulmonary hypertension or cardiac dysfunction are difficult to predict given the differences between the fetal and the neonatal circulation.

The immediate postpartum period provides clinicians a first glance of the infant's respiratory capacity. Consequently, this period also enables clinicians a chance to determine the severity of the congenital abnormality by monitoring physiological parameters and/or ventilatory requirements. There are already several scoring systems to determine the risk of adverse outcomes available, such as the Score for Neonatal Acute Physiology–II (SNAP-II) score, Wilford Hall/Santa Rosa prediction model and the Brindle scoring model (36–40). Most of these scoring systems are combining several clinical parameters (such as blood pressure, serum pH, fraction of inspired oxygen FiO_2_) yet the majority of these scoring systems use the worst values of the parameters within the first 12–24 h (37–39).

We speculate that the level of respiratory support required during the initial neonatal resuscitation may provide important information of possible adverse outcomes. For instance, as described above, the need to supplement with a high FiO_2_ concentration in the delivery room appears to be associated with higher morbidity and mortality for CDH infants (13, 41). Likewise, similar observations were reported regarding expiratory tidal volumes, end-tidal carbon dioxide levels and dynamic lung compliance (41, 42). Respiratory monitors now allow real-time measurements of several lung function parameters. Recording these parameters gives an opportunity to gather large datasets by aggregating individual patient data within a framework of multicenter collaborations, which can be used for prediction modeling to identify early signs of deterioration. In addition, combining physiological and ventilatory outcome measures, such as is done with the oxygen saturation index (OSI), may improve the predictive value of these models for adverse models even further (36).

## Conclusion

Herein, we have described some of the trending new concepts regarding interventions in the early perinatal stabilization phase for infants with CDH. Our understanding of the physiological adaptations immediately after birth has certainly grown in recent decades, but considerable knowledge gaps are remaining. Regardless, thorough investigation using appropriate preclinical models is essential prior to translating novel concepts into clinical practice. Importantly, optimizing the fetal to neonatal transition will not only improve postnatal outcomes for infants born with CDH, but also for those born with abnormal lung development caused by a broad range of other conditions, such as prolonged anhydramnios.

## Author Contributions

PD and IR were involved in the conception of this paper and wrote the first draft, which was critically reviewed by all authors. The final version was approved by all authors.

## Funding

PD and EH-O were supported by a grant from Sophia Children's Hospital Foundation (SSWO, Grant S19-12). This research was supported by the National Health and Medical Research Council (NHMRC) Project Grant APP1187580 (KC and PD).

## Conflict of Interest

The authors declare that the research was conducted in the absence of any commercial or financial relationships that could be construed as a potential conflict of interest.

## Publisher's Note

All claims expressed in this article are solely those of the authors and do not necessarily represent those of their affiliated organizations, or those of the publisher, the editors and the reviewers. Any product that may be evaluated in this article, or claim that may be made by its manufacturer, is not guaranteed or endorsed by the publisher.
